# The Story of the Insane from Year to Year

**Published:** 1902-01-04

**Authors:** 


### THE STORY OF THE INSANE FROM YEAR TO YEAR.

(THIRTEENTH SERIES.)

### THE EDINBURGH ROYAL ASYLUM.

ON January 1st, 1899, this asylum contained 945 patients, and on December 31st the number had fallen to 912, this diminution having been caused by the transference of patients to the asylums at Hartwood and Lambert. There were 323 first admissions, 105 readmissions, 172 recoveries, 84 discharged relieved, 100 discharged not improved, and 105 deaths. The average number resident during 1899 was 907, and the total number under treatment was 1,346. Calculated on the admissions, the recoveries stand at 35 per cent., and calculated on the total number resident, the deaths stand at 7·7 per cent.; but if the method of calculation usual in asylums be adopted the latter percentage would be 11·5. The recoveries are rather below the usual asylum average, and deaths are rather higher. Of the year's deaths 52 were caused by cerebral and spinal diseases, 13 by consumption, and 5 by pneumonia. Of the year's admissions we notice that various moral causes are supposed to have been instrumental in producing the insanity in 27 instances, while of the physical causes, "previous attacks" comes first with 121, hereditary influence next with 109, and intemperance in drink with 88. Unfortunately, any value this table may have possessed is considerably lessened by the fact that in 192 of the 428 admissions, no cause was known. Referring to the production of insanity by alcoholic excess, Dr. Clouston says, "I wish I could report even the sure beginning of a slow improvement among our population in this matter." And, "it is precisely the men and women to whose brains it is most dangerous, who so frequently take it to excess." Dr. Clouston thinks, and we believe him to be right in so thinking, that the Inebriates Act of 1898 was generally colourless and limited in scope, and that it gave rise to keen disappointment. It is indeed that utterly false cry, "the liberty of the subject," that has done, and will continue to do, so much harm in this, and some other matters too, until public opinion can be educated up to the point when it can distinguish between "license" and "liberty." The only ways to materially lessen the amount of insanity in the world are, as Dr. Clouston truly says, to live according to physiological and moral law, and to arrange suitable marriages. The managers say they "have again to record their sense of obligation to Dr. Clouston for his indefatigable labours to promote the interests of the Institution." The cost per head of the New Craig House patients was £117 per annum; that of the intermediate patients was £40; and that of the paupers £32.

